# Neuropsychiatric adverse events associated with Glucagon-like peptide-1 receptor agonists: a pharmacovigilance analysis of the FDA Adverse Event Reporting System database

**DOI:** 10.1192/j.eurpsy.2024.1803

**Published:** 2025-02-04

**Authors:** Wenchao Lu, Shihan Wang, Huilin Tang, Tao Yuan, Wei Zuo, Yuling Liu

**Affiliations:** 1 Institute of Materia Medica, Chinese Academy of Medical Sciences and Peking Union Medical College, Beijing, China; 2 Department of Pharmacy, Peking Union Medical College Hospital, Chinese Academy of Medical Sciences and Peking Union Medical College, Beijing, China; 3 Department of Endocrinology, Key Laboratory of Endocrinology of Ministry of Health, Peking Union Medical College Hospital, Chinese Academy of Medical Sciences and Peking Union Medical College, Beijing, China; 4 Department of Pharmaceutical Outcomes and Policy, University of Florida College of Pharmacy, Gainesville, FL, USA

**Keywords:** FAERS, GLP-1RAs, neuropsychiatric adverse events, pharmacovigilance analysis

## Abstract

**Background:**

Glucagon-like peptide-1 receptor agonists (GLP-1RAs) are widely used due to their profound efficacy in glycemic control and weight management. Real-world observations have revealed potential neuropsychiatric adverse events (AEs) associated with GLP-1RAs. This study aimed to comprehensively investigate and characterize these neuropsychiatric AEs with GLP-1RAs.

**Methods:**

We analyzed GLP-1RA adverse reaction reports using the FDA Adverse Event Reporting System database. Disproportionality analysis using reporting odds ratio (ROR) identified eight categories of neuropsychiatric AEs associated with GLP-1RAs. We conducted descriptive and time-to-onset (TTO) analyses and explored neuropsychiatric AE signals among individual GLP-1RAs for weight loss and diabetes mellitus (DM) indications.

**Results:**

We identified 25,110 cases of GLP-1RA-related neuropsychiatric AEs. GLP-1RAs showed an association with headache (ROR 1.74, 95% confidence interval [CI] 1.65–1.84), migraine (ROR 1.28, 95%CI 1.06–1.55), and olfactory and sensory nerve abnormalities (ROR 2.44, 95%CI 1.83–3.25; ROR 1.69, 95%CI 1.54–1.85). Semaglutide showed a moderate suicide-related AEs signal in the weight loss population (ROR 2.55, 95%CI 1.97–3.31). The median TTO was 16 days (interquartile range: 3–66 days).

**Conclusions:**

In this study, we identified eight potential neuropsychiatric adverse events (AEs) associated with GLP-1RAs and, for the first time, detected positive signals for migraine, olfactory abnormalities, and sensory abnormalities. We also observed positive suicide-related signals of semaglutide, in weight loss population. This study provides a reliable basis for further investigation of GLP-1RA-related neuropsychiatric AEs. However, as an exploratory study, our findings require confirmation through large-scale prospective studies.

## Background

Obesity and diabetes mellitus (DM) have become significant burdens of global public health. It is estimated that more than half of the world’s population will be diagnosed as overweight or obese by the year 2035 [1]; over 1.31 billion individuals will be affected by DM, and type 2 diabetes mellitus (T2DM) makes up the vast majority [2].

Glucagon-like peptide-1 receptor agonists (GLP-1RAs) can manage T2DM and obesity by stimulating glucose-dependent insulin, suppressing postprandial glucagon, inhibiting gastric emptying, reducing appetite and food intake, and protecting islet cell function [3]. The U.S. Food and Drug Administration (FDA) and the European Medicines Agency (EMA) have approved GLP-1RAs for the therapy of T2DM in 2005 and 2006, and for obesity in 2014 and 2015, respectively. After that, liraglutide, dulaglutide, and semaglutide were approved for T2DM with major cardiovascular adverse events (MACEs). In March 2024, semaglutide was the first anti-obesity drug approved for overweight or obese adults with MACEs [4]. In addition to the approved indications mentioned above, a growing body of research supports the metabolic regulatory effects of GLP-1RAs, such as weight loss, improvement of insulin resistance, regulation of sex hormone levels, improvement of blood lipid profiles, and reduction of hepatic steatosis [5, 6]; therefore, GLP-1RAs are gradually showing beneficial effects in endocrine disorders or metabolic diseases such as polycystic ovary syndrome, lean nonalcoholic fatty liver disease, and obstructive sleep apnea [7], thus making GLP-1RAs one of the most popular and anticipated drug stars today.

Indeed, the increasing utilization of GLP-1RAs has provided us with a more profound comprehension of their adverse effects (AEs). In addition to commonly observed gastrointestinal AEs, particular attention was given to potential risks such as pancreatic cancer, thyroid cancer, cholelithiasis, hepatotoxicity, acute kidney injury, increased heart rate, angioedema, and injection site reactions [3, 8]. When it comes to neuropsychiatric AEs, some case reports mention that the clinical application of exenatide, semaglutide, and liraglutide may be related to the onset or recurrence of depression [9–11]. In July 2023, the Icelandic Medicines Agency reported that patients treated with liraglutide and semaglutide were at possible risk of self-harm and suicide; thus, EMA soon announced a formal review of related risks with GLP-1RAs [12]. Concurrently, potential new safety concerns for GLP-1RAs, including alopecia, aspiration, and suicidal ideation, were identified within the FDA Adverse Event Reporting System (FAERS) database [13]. Also, the knowledge of neurological side effects for GLP-1RAs is limited, with only dizziness and taste disturbances mentioned in the drug instructions [14]. To the best of our knowledge, natural glucagon-like peptide-1 (GLP-1) is secreted by preproglucagon neurons in the central nervous system (CNS) [15]. Among patients with obesity and T2DM, the blood–brain barrier is dynamically altered or even impaired. Liraglutide, exenatide, and dulaglutide have been shown to rapidly cross the blood–brain barrier by passive diffusion, and tirzepatide crosses the blood–brain barrier slowly, presumably by extracellular pathways [16–18]. Semaglutide cannot cross the normal blood–brain barrier directly but can bind to serum albumin and is taken up by Tanycyte cells through the ventricular walls of the CNS [19]. GLP-1RAs act directly or indirectly on GLP-1 receptors of the CNS to mediate reduced energy intake, increased satiety, facilitated insulin signaling, and other central effects. Given the emerging concerns about psychiatric effects and the gaps in our understanding of neurological impacts, it is crucial to investigate the neuropsychiatric side effects of GLP-1RAs more thoroughly.

In clinical practice, individuals with obesity and T2DM are at an increased risk of experiencing neuropsychiatric abnormalities and may require related medication [20–25]. Therefore, it is crucial to consider the potential neuropsychiatric side effects when selecting appropriate antidiabetic medications. Furthermore, despite some clinical studies examining the psychiatric AEs of GLP-1RAs [26], their inclusion criteria and follow-up time limitations make it challenging for them to fully capture the neuropsychiatric effects of GLP-1RA drugs on obese and diabetic populations in real-world settings. FAERS is a comprehensive and openly accessible pharmacovigilance database containing real-world adverse drug reactions (ADRs) recorded by the FDA [27, 28], which serves as a valuable tool for assessing ADRs in the post-marketing phase.

In this study, we aim to update and broaden the understanding of psychiatric AEs linked to GLP-1RAs while also exploring their neurological AEs for the first time. We examine differences among various GLP-1RAs and indications. Furthermore, we compile reports on specific neuropsychiatric AEs associated with other antidiabetic and anti-obesity medications to provide improved clinical guidance for drug management.

## Methods

### Data source

We conducted a pharmacovigilance study on neuropsychiatric AEs associated with GLP-1RAs from the FAERS database, a publicly available database of safety reports submitted by patients, healthcare professionals, and pharmaceutical companies [27]. We downloaded the FAERS data files from 2010Q1 to 2024Q1. We used generic and brand names to identify GLP-1RAs, including exenatide (BYETTA, BYDUREON), liraglutide (VICTOZA, SAXENDA), dulaglutide (TRULICITY), semaglutide (OZEMPIC, RYBELSUS, WEGOVY), and tirzepatide (MOUNJARO). Only cases with “primary suspected (PS)” use of GLP-1RAs were included. The generic and brand names of all GLP-1RA, anti-obesity, or antidiabetic medications are included in Supplementary Figure S1.

Additionally, the Medical Dictionary for Regulatory Activities (MedDRA) Preferred Term (PT) codes are systematically used to code the adverse reactions that are documented in the FAERS database [28]. With PTs acting as distinctive identifiers for particular medical ideas, such as symptoms, signs, and illness diagnoses, this lexicon is arranged into five distinct hierarchical levels. The hierarchy further classifies the medical ideas by including “High-Level Terms” (HLTs) and “High-Level Group Terms” (HLGTs) in addition to PTs. Ultimately, based on their genesis, presentation place, or intended purpose, these HLGTs are classified into “Systemic Organ Classes” (SOCs). Different PTs can be organized into discrete SOCs because of its multiaxial structure, with a principal SOC assigned to each classification.

Utilizing this framework, we focused our analysis on neuropsychiatric PTs associated with “psychiatric disorders” and “nervous system disorders” as the primary SOC. Specifically, we retrieved PTs corresponding to all psychiatric AEs (*N* = 564) and nervous AEs (*N* = 1,059) from MedDRA (version 24.1).

### Data processing procedure

We deduplicated the reports of GLP-1RAs obtained from the FAERS database, and in the deduplication process, we extracted the latest (most recent) case version from all available cases based on the case ID, case initial/follow-up code (“I” or “F”), case event date, age, sex, and reporting country. We retained the most current case version and removed all others [29].

### Descriptive analysis and time-to-onset analysis

We performed a descriptive analysis of the clinical characteristics of reports with GLP-1RA-related neuropsychiatric AEs after screening, including sex, age, age group, reporter type, report year, indication, outcome, and the groups of time-to-onset (TTO). TTO was defined as the interval between the initiation of GLP-1RAs and the occurrence of an AE. When calculating the onset time, we only selected data with an onset time greater than 0 days. Reports with incorrect dates (i.e., time of dosing later than the time of the event) and missing dates were not included.

### Disproportionality analysis

In pharmacovigilance studies, disproportionality analysis primarily evaluates possible associations between a specific AE and a particular drug. Reporting odds ratio (ROR) compares the odds of reporting an event of interest in a specific drug to all other events relative to the reporting odds for other drugs in the FAERS database [30]. Neuropsychiatric adverse reaction signal was considered valid and associated with GLP-1RA treatment if the number of reports of neuropsychiatric AEs was not less than 3 and the lower limit of the 95% confidence interval (CI) of the ROR exceeded 1 [30]. The following formula was used to calculate the ROR and 95% CI:

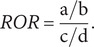

**Table:** 

	GLP-1RAs	All other drugs in the database
Neuropsychiatric adverse events	*a*	*c*
All other adverse events in the database	*b*	*d*

Where *a* is the number of neuropsychiatric AEs cases involving GLP-1RAs, *b* is the number of all other AE case reports involving GLP-1RAs, *c* is the number of neuropsychiatric AE cases involving all other drugs in the whole database, and *d* is the number of other AE cases involving all other drugs in the whole database.

In the disproportionality analysis, first, a comprehensive assessment of GLP-1RA-related neuropsychiatric AEs was conducted based on DM and weight control indications. Subsequently, according to MedDRA, we selected eight significant neuropsychiatric signals and aggregated the related AE groups at the HLT/HLGT level. This approach ensured that the PTs analyzed were clinically relevant and accurately represented actual psychiatric and nervous AEs, thus strengthening the validity of our subsequent analysis. Then, as for the selected neuropsychiatric AEs, we conducted a disproportionality analysis by comparing every individual GLP-1RA with non-GLP-1RA anti-obesity or antidiabetic medications. This information can be a valuable reference for healthcare professionals and patients when selecting appropriate medications. Finally, the relevant signals from other drug classes with similar indications were examined to compare GLP-1RAs with other medicines for the same indication; we additionally collected reports of the specific neuropsychiatric AEs associated with other antidiabetic and anti-obesity medications.

## Results

### Descriptive analysis

**Figure 1.**
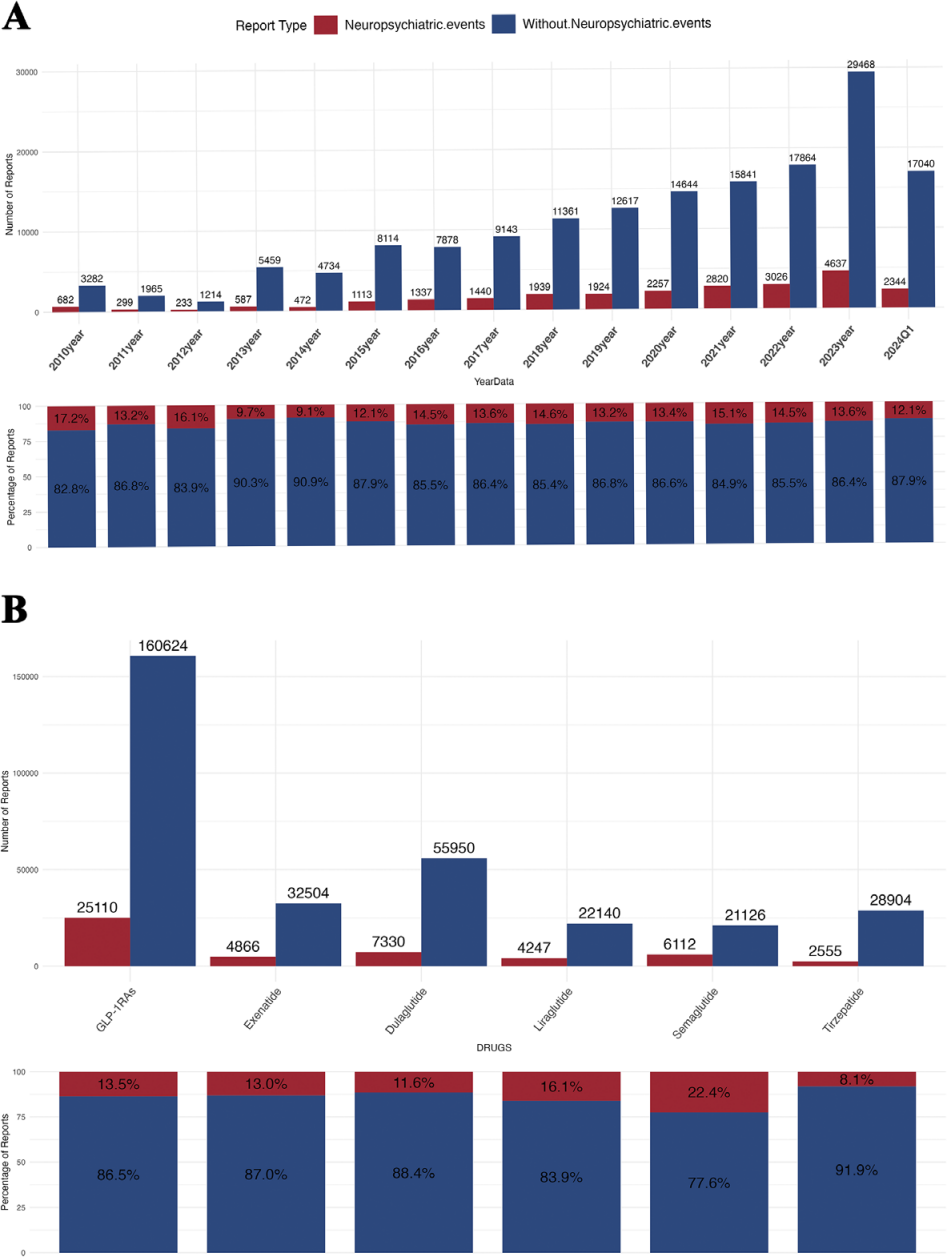
Data on reported cases of glucagon-like peptide-1 receptor agonist (GLP-1RA)-related neuropsychiatric adverse events (AEs) from the FDA Adverse Event Reporting System (FAERS) database between January 1, 2010 and March 31, 2024 (2010Q1–2024Q1). (A) The upper bar plot depicts the number of GLP-1RA reports with neuropsychiatric AEs versus those without for each year in the FAERS database from 2010Q1 to 2024Q1. The proportional bar chart below illustrates the ratio of GLP-1RA reports with neuropsychiatric AEs compared to those without for each year in the FAERS database during 2010Q1–2024Q1. Red denotes reports with neuropsychiatric AEs, while blue denotes those without. (B) The upper bar plot presents the number of GLP-1RA reports involving neuropsychiatric adverse reactions versus those without for various GLP-1RA treatment strategies in the FAERS database from 2010Q1 to 2024Q1. The proportional bar chart below shows the proportion of GLP-1RA reports with neuropsychiatric AEs compared to those without for different GLP-1RAs in the FAERS database from 2010Q1 to 2024Q1.

As depicted in Figure 1, the number of AE reports associated with GLP-1RAs exhibited a consistent annual increase from 2016 to 2023. The number of reports witnessed a dramatic surge, reaching 34,105 in 2023. Furthermore, there was a dramatic rise in neuropsychiatric AEs reports from 1,924 cases in 2019 to 4,637 cases in 2023. Among individual GLP-1RAs, dulaglutide had the highest reported overall AEs (63,280 cases, 34.1%) and neuropsychiatric AEs (7,330 cases, 29.2%). Notably, tirzepatide, launched in 2022, had the third-highest overall reported AEs (31,459 cases, 16.9%). The proportion of neuropsychiatric/overall reported AEs was highest for semaglutide (22.4%) among various GLP-1RAs drugs.

From 2010 to 2024Q1, 25,110 cases of GLP-1RA-related neuropsychiatric AEs were identified, comprising 15,860 females (63.2%) and 7,920 males (31.5%). Most reported adverse reactions occurred in individuals aged between 18 and 65, accounting for 33.3%, followed by those aged between 65 and 85, accounting for 20.1%. The proportion of patients under the age of 18 and over 85 was relatively low, both below 1%. Regarding the indications, DM, obesity, and missing information accounted for 54.4%, 8.5%, and 35.8%, respectively. The data on reported outcomes were missing in 63% of the cases; hospitalization was observed in 11.8% of adverse reaction cases, and life-threatening or fatal outcomes accounted for 1.5% (Table 1).

**Table 1.** Characteristics of reports with GLP-1RA-related neuropsychiatric adverse events sourced from the FDA Adverse Event Reporting System database (January 1, 2010 to March 31, 2024)

	GLP–1RA*N* (%)	Exenatide*N* (%)	Liraglutide*N* (%)	Dulaglutide*N* (%)	Semaglutide*N* (%)	Tirzepatide*N* (%)
	(*N* = 25,110)	(*N* = 4,866)	(*N* = 4,247)	(*N* = 7,330)	(*N* = 6,112)	(*N* = 2,555)
Sex						
F	15,860 (63.2%)	3,083 (63.4%)	2,879 (67.8%)	4,245 (57.9%)	3,940 (64.5%)	1,713 (67.0%)
M	7,920 (31.5%)	1,646 (33.8%)	1,290 (30.4%)	2,557 (34.9%)	1,902 (31.1%)	525 (20.5%)
Missing	1,330 (5.3%)	137 (2.8%)	78 (1.8%)	528 (7.2%)	270 (4.4%)	317 (12.4%)
Age						
<18 years	28 (0.1%)	1 (0.0%)	14 (0.3%)	4 (0.1%)	8 (0.1%)	1 (0.0%)
>85 years	136 (0.5%)	20 (0.4%)	13 (0.3%)	60 (0.8%)	38 (0.6%)	5 (0.2%)
18–64.9 years	8,363 (33.3%)	1,251 (25.7%)	1,978 (46.6%)	1,644 (22.4%)	2,234 (36.6%)	1,256 (49.2%)
65–85 years	5,049 (20.1%)	942 (19.4%)	975 (23.0%)	1,395 (19.0%)	1,422 (23.3%)	315 (12.3%)
Missing	11,534 (45.9%)	2,652 (54.5%)	1,267 (29.8%)	4,227 (57.7%)	2,410 (39.4%)	978 (38.3%)
Reporter						
Healthcare professional	4,216 (16.8%)	609 (12.5%)	1,143 (26.9%)	672 (9.2%)	1,633 (26.7%)	159 (6.3%)
Non-healthcare professional	19,743 (78.6%)	3,227 (66.3%)	3,045 (71.7%)	6,633 (90.5%)	4,448 (72.8%)	2,390 (93.5%)
Unknown or missing	1,151 (4.6%)	1,030 (21.2%)	59 (1.4%)	25 (0.3%)	31 (0.5%)	6 (0.2%)
Report year						
2010	682 (2.7%)	262 (5.4%)	420 (9.9%)	0 (0%)	0 (0%)	0 (0%)
2011	299 (1.2%)	103 (2.1%)	196 (4.6%)	0 (0%)	0 (0%)	0 (0%)
2012	233 (0.9%)	155 (3.2%)	78 (1.8%)	0 (0%)	0 (0%)	0 (0%)
2013	587 (2.3%)	335 (6.9%)	252 (5.9%)	0 (0%)	0 (0%)	0 (0%)
2014	472 (1.9%)	363 (7.5%)	107 (2.5%)	2 (0.0%)	0 (0%)	0 (0%)
2015	1,113 (4.4%)	501 (10.3%)	448 (10.5%)	164 (2.2%)	0 (0%)	0 (0%)
2016	1,337 (5.3%)	426 (8.8%)	490 (11.5%)	421 (5.7%)	0 (0%)	0 (0%)
2017	1,440 (5.7%)	501 (10.3%)	315 (7.4%)	624 (8.5%)	0 (0%)	0 (0%)
2018	1,939 (7.7%)	524 (10.8%)	530 (12.5%)	761 (10.4%)	124 (2.0%)	0 (0%)
2019	1,924 (7.7%)	495 (10.2%)	251 (5.9%)	925 (12.6%)	253 (4.1%)	0 (0%)
2020	2,257 (9.0%)	422 (8.7%)	277 (6.5%)	1,001 (13.7%)	557 (9.1%)	0 (0%)
2021	2,820 (11.2%)	352 (7.2%)	303 (7.1%)	1,344 (18.3%)	821 (13.4%)	0 (0%)
2022	3,026 (12.1%)	222 (4.6%)	231 (5.4%)	1,091 (14.9%)	1,163 (19.0%)	319 (12.5%)
2023	4,637 (18.5%)	161 (3.3%)	245 (5.8%)	812 (11.1%)	2,100 (34.4%)	1,319 (51.6%)
2024Q1	2,344 (9.3%)	44 (0.9%)	104 (2.4%)	185 (2.5%)	1,094 (17.9%)	917 (35.9%)
Indication						
Diabetes mellitus or blood glucose abnormal	12,158 (54.35%)	3,327 (68.37%)	1,691 (39.82%)	3,898 (53.18%)	2,259 (36.96%)	1,034 (40.47%)
Weight control	1,909 (8.53%)	29 (0.60%)	567 (13.35%)	49 (0.67%)	901 (14.74%)	298 (11.66%)
Others	288 (1.29%)	60 (1.23%)	45 (1.06%)	19 (0.26%)	97 (1.59%)	37 (1.45%)
Unknown or missing	8,013 (35.82%)	1,450 (29.80%)	1,944 (45.77%)	3,364 (45.89%)	2,855 (46.71%)	1,186 (46.42%)
Outcome						
Death	250 (0.9%)	78 (1.4%)	56 (1.2%)	67 (0.9%)	39 (0.6%)	10 (0.4%)
Disability	435 (1.6%)	68 (1.2%)	79 (1.7%)	109 (1.4%)	148 (2.3%)	31 (1.2%)
Hospitalization	3,181 (11.8%)	845 (15.4%)	647 (14.1%)	859 (11.1%)	684 (10.5%)	146 (5.5%)
Life-threatening	406 (1.5%)	113 (2.1%)	90 (2.0%)	74 (1.0%)	110 (1.7%)	19 (0.7%)
Congenital anomaly	4 (0.1%)	1 (0.0%)	1 (0.0%)	1 (0.0%)	0 (0%)	1 (0.0%)
Other serious	5,574 (20.7%)	1,257 (23.0%)	1,095 (23.8%)	1,542 (20.0%)	1,529 (23.3%)	280 (10.7%)
Missing	16,982 (63.0%)	3,112 (56.9%)	2,633 (57.2%)	5,060 (65.6%)	4,029 (61.6%)	2,148 (81.5%)
The time to onset (days)						
0–30	1,804	253	406	444	555	146
31–60	347	50	66	46	161	24
61–90	164	16	24	26	81	17
91–120	111	13	18	11	56	13
121–150	83	10	10	9	48	6
151–180	57	8	11	7	23	8
181–360	153	28	34	23	55	13
>360	219	101	55	25	32	6

Abbreviations: F, female; GLP-1RA: glucagon-like peptide-1 receptor agonist; M, male.

### Disproportionality analysis

**Figure 2.**
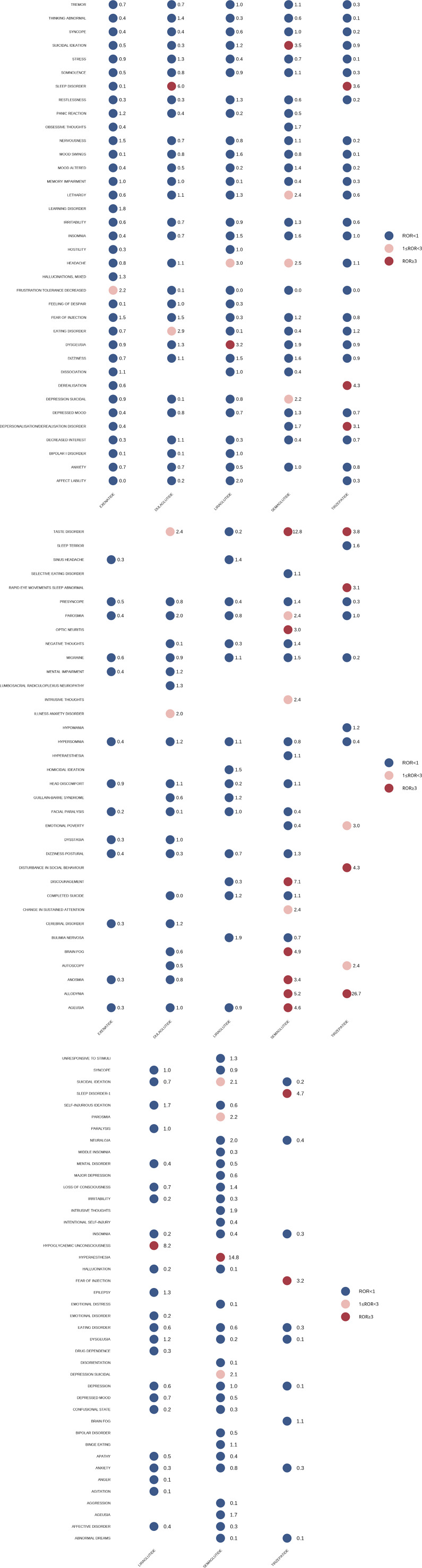
Scanning for Glucagon-like peptide-1 receptor agonist (GLP-1RA)-related neuropsychiatric adverse events (AEs) based on the diabetes indication and weight loss indication in the FDA Adverse Event Reporting System (FAERS) database. (A) Diabetes indication cohort. (B) Weight loss indication cohort. The heatmap shows the lower limit of the 95% confidence interval for the reporting odds ratio (ROR [RORL]) for neuropsychiatric AEs (with cases no less than 3) in the FAERS database under different GLP-1RAs. Dark red indicates RORL values greater than 3, and light red indicates RORL values less than 3 and greater than 1; dark blue indicates RORL values less than 1; white indicates RORL values that could not be calculated. Neuropsychiatric AEs labeled with red or blue color meet the criteria that the number of cases occurring no less than 3.

**Figure 3.**
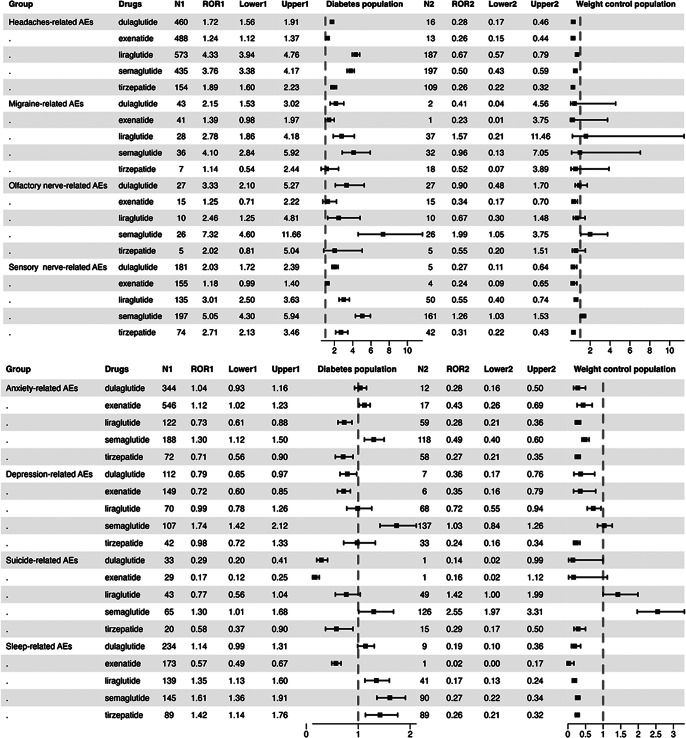
Forest plot showing the reporting odds ratio (ROR) of eight specific neuropsychiatric adverse events with different glucagon-like peptide-1 receptor agonists based on diabetes indication population and weight loss indication population. N1, ROR1, Lower1, and Upper1 are data related to the diabetes population, whereas N2, ROR2, Lower2, and Upper2 are data related to the weight control population.

The neuropsychiatric AE signals were identified based on the indications of GLP-1RAs. As shown in Figure 2, GLP-1RAs exhibited strong adverse reaction signals for anxiety, suicide, sleep disturbances, headaches, sensory nerve abnormalities, olfactory nerve abnormalities, and migraines. Consequently, we conducted an aggregated analysis of these AEs according to HLGT/HLT levels and performed disproportionality analysis for each adverse reaction group. As can be seen from Figure 3, comparing to non-GLP-1RA antidiabetic medications, all five GLP-1RAs were found to be associated with headache in patients with DM: liraglutide (ROR 4.33, 95%CI 3.94–4.76) and semaglutide (ROR 3.76, 95%CI 3.38–4.17) showed the stronger signals; dulaglutide, liraglutide, and semaglutide exhibited a significant relationship with migraines, with semaglutide demonstrating the strongest association (ROR 4.10, 95%CI 2.84–5.92); dulaglutide, liraglutide, and semaglutide showed olfactory nerve disorder signals, and semaglutide demonstrated the strongest signal (ROR 7.32, 95%CI 4.60–11.66), followed by dulaglutide (ROR 3.33, 95%CI 2.10–5.27); sensory nerve abnormalities were associated with dulaglutide, liraglutide, semaglutide, and tirzepatide, and among these drugs, semaglutide showed the strongest signal (ROR 5.05, 95%CI 4.30–5.94) and the most significant number of reports (*N* = 197), followed by liraglutide (ROR 3.01, 95%CI 2.50–3.63). Weak anxiety signals were observed only in exenatide (ROR 1.12, 95%CI 1.02–1.23) and semaglutide (ROR 1.30, 95%CI 1.12–1.50); most of the GLP-1RAs were not associated with suicide-related AEs, except that Semaglutide showed a very weak signal (ROR 1.30, 95%CI 1.01–1.68) in patients with DM. Notably, semaglutide detected a moderate suicide signal(ROR 2.55, 95%CI 1.97–3.31) and liraglutide showed very weak signal (ROR 1.42, 95%CI 1.00-1.99) in the weight loss population; sleep disorders were associated with liraglutide, semaglutide, and tirzepatide in diabetic patients but not in weight control patients. In weight control patients, compared to non-GLP-1RA anti-obesity medications, individual GLP-1RAs were not observed signals among the above AEs except for semaglutide (Figure 3).

### Neuropsychiatric AE profiles with other drugs based on indication

**Figure 4.**
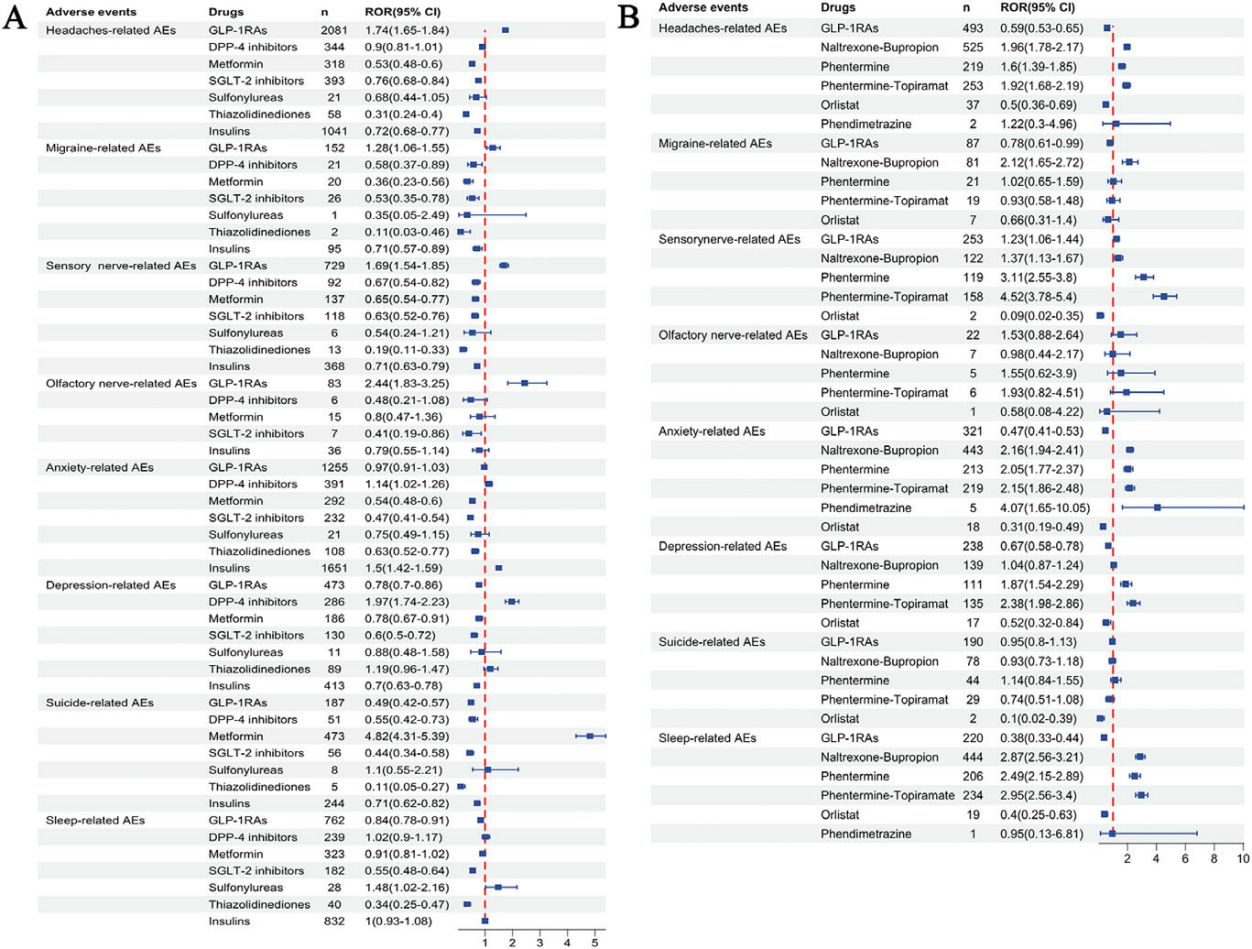
Forest plot showing the reporting odds ratio (ROR) of eight specific neuropsychiatric adverse events (AEs) with different drugs. (A) The ROR of different glucose-lowering-drug-related neuropsychiatric AEs. (B) The ROR of different weight-loss-drug-related neuropsychiatric AEs.

We conducted additional analyses to evaluate the neuropsychiatric AE profiles with other medications used for the same indication (Figure 4). Among the classes of medicines for DM, only GLP-1RAs showed an association with headache (ROR 1.74, 95%CI 1.65–1.84), migraine (ROR 1.28, 95%CI 1.06–1.55), and olfactory and sensory nerve abnormalities (ROR 2.44, 95%CI 1.83–3.25; ROR 1.69, 95%CI 1.54–1.85). Sulfonylureas (ROR 1.48, 1.02–2.16) showed a weak signal risk for sleep-related adverse reactions, whereas other antidiabetic drugs did not exhibit relevant signal risks. For the outcomes of anxiety, depression, and suicide, GLP-1RAs demonstrated no overall correlation signal; dipeptidyl peptidase-4 (DPP-4) inhibitors displayed signals related to anxiety (ROR 1.14, 95%CI 1.02–1.26) and depression (ROR 1.97, 95%CI 1.74–2.23); interestingly, metformin exhibited a moderate suicide signal (ROR 4.82, 95%CI 4.31–5.39). When used in the weight-loss population, GLP-1RAs only showed a weak signal for sensory nerve abnormalities (ROR 1.23, 95%CI 1.06–1.44) without any significant signals in the other seven specific neuropsychiatric AEs. Three commonly used anti-obesity medications – naltrexone-bupropion, phentermine, and phentermine-topiramate – were associated with headaches, insomnia, sensory nerve abnormalities, and anxiety. None of the anti-obesity medications showed any signals related to suicide. Furthermore, no signals were found for orlistat regarding the eight specific neuropsychiatric AEs mentioned above (Figure 4).

### The time-to-onset analysis

**Figure 5.**
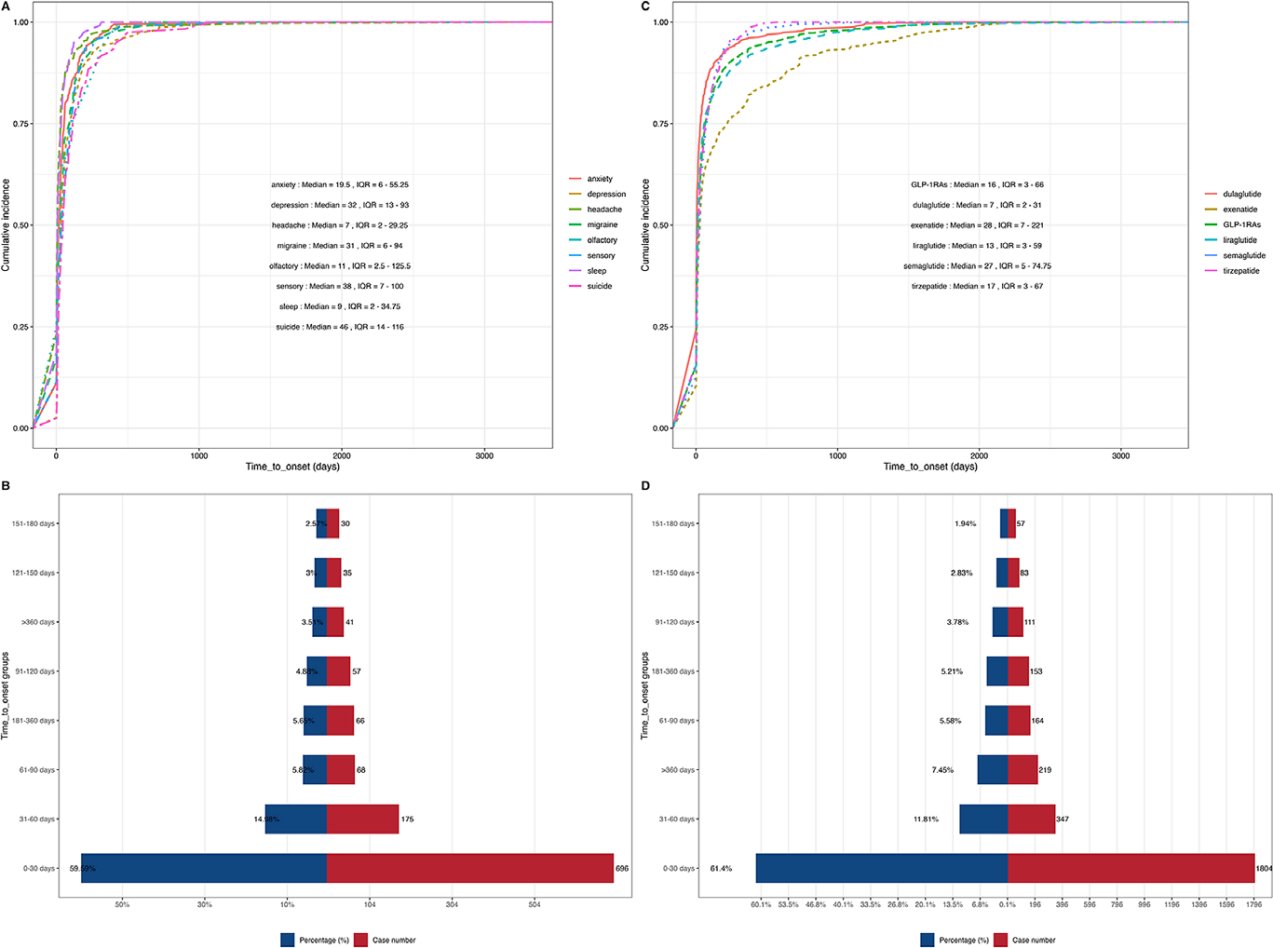
The time-to-onset (TTO) analysis of glucagon-like peptide-1 receptor agonist glucagon-like peptide-1 receptor agonist (GLP-1RA)-related neuropsychiatric adverse events (AEs). (A) Cumulative distribution curves demonstrating the TTO of eight specific neuropsychiatric AEs of GLP-1RAs. (B) The percentage and number of different TTO groups for eight specific neuropsychiatric AEs of GLP-1RAs. (C) Cumulative distribution curves demonstrating TTO of all GLP-1RA-associated neuropsychiatric AEs. (D) The percentage and number of different TTO groups for all GLP-1RA-associated neuropsychiatric AEs.

The TTO analysis of eight specific neuropsychiatric AEs associated with GLP-1RAs revealed that the majority occurred within 30 days of GLP-1RAs administration. Among these particular neuropsychiatric AEs, it was noteworthy that the earliest median time of TTO was observed for headache-related AEs (median = 7 days, interquartile range [IQR] = 2–29.25 days), followed by sleep-related adverse reactions (median = 9 days, IQR = 2–34.75 days) and olfactory nerve disorders (median = 11 days, IQR = 2.5–125.5 days). Suicide-related adverse reactions exhibited a later median onset time (median = 46 days, IQR =14–116 days), preceded by sensory nerve disorders (median = 38 days, IQR = 7–100 days) and depression symptoms (median = 32 days, IQR = 13–93 days). In terms of individual GLP-1RAs, which showed variations in their respective median time, exenatide had the longest median TTO period (median = 28 days, IQR = 7–221 days), followed closely by semaglutide (median = 27 days, IQR = 5–74.75 days). In contrast, dulaglutide demonstrated one of the shortest durations (median = 7 days, IQR = 2–31 days) (Figure 5).

## Discussion

As far as we know, this study is the first comprehensive pharmacovigilance investigation into the potential AEs of neuropsychiatric systems for GLP-1RAs through real-world data from the FAERS database. First, we detailed the clinical characteristics of reported cases of GLP-1RA-associated neuropsychiatric ADRs. Second, we grouped DM and weight-loss populations according to the main indications of GLP-1RAs and identified eight potential neuropsychiatric ADRs related to GLP-1RAs using disproportionate analysis. Then, we explored the specific effects of each drug of GLP-1RAs and other antidiabetic and anti-obesity medications on neuropsychiatric ADRs.

GLP-1RA-related neuropsychiatric AEs accounted for 13.5% among all the cases of GLP-1RAs in the FAERS database from 2010 to 2024Q1, and it showed an uptrend from 2015 to 2023, reflecting more and more concerns about GLP-1RA-related psychoneurological AEs. This was partly attributable to the fact that expanded indications, aggressive marketing by influencers, and high media exposure of GLP-1RAs in recent years contributed to more and more prescriptions [31]. Another explanation can be that the growing interest in their neuropsychiatric AEs in recent years encourage related declaration by clinicians and patients. Concerning suicidal risk with GLP-1RAs can also be link to prescription biases [32]. In addition, this may be due to the black swan event that the novel coronavirus pneumonia epidemic that began in late 2019 has exerted a significant impact on the physical and mental health of global population [33]. Consistent with previous studies [34, 35], semaglutide (22.4%) had a relatively higher proportion of neuropsychiatric AEs compared to other GLP-1RA medications, which may be attributed to its large market sales and the high level of concern about its safety in mental illness [31]. In addition, one study suggested that semaglutide was associated with drug abuse, prescription drug use without prescriptions, and intentional use [36], which also increased its proportion of neuropsychiatric AEs.

In terms of gender distribution, the proportion of females (63.2%) reporting ADR cases was significantly higher than that of males (31.5%), consistent with prior studies [34, 35, 37, 38]. This may be because a higher proportion of female patients who receive treatment of GLP-1RAs suffer neuropsychiatric abnormalities than males, especially in the obese population [39, 40], and prefer to share their discomfort symptoms. In the age group, except for missing data (45.9%), adults aged 18–65 made up the majority of reported ADRs (33.3%), and the proportion of patients under 18 years old and over 85 years old was less than 1%. Studies suggested that adult patients exhibited higher susceptibility to self-injurious and suicidal tendencies compared to other age groups [33]. In clinical practice, clinicians are more cautious about prescribing GLP-1RAs for pediatric and elderly patients. Furthermore, compared to adult patients, adolescents and the elderly lack related consciousness, tools, and skills to report ADRs.

Regarding the TTO of overall GLP-1RA neuropsychiatric ADRs, the median time was 16 days (IQR = 3–66 days). This suggested that close attention should be paid to neuropsychiatric feedback from initial medication. Among these GLP-1RAs, the TTO of dulaglutide was the shortest at 7 days, while exenatide was the longest at 28 days, parallel to previous pharmacovigilance studies [34]. We speculate that differences in TTO among GLP-1RAs drugs may be due to their various pharmacokinetics and the action mechanism with the nervous system. Moreover, we further found that the proportion of neuropsychiatric ADRs of GLP-1RAs overall decreased with the duration of application; this suggested that like gradual tolerance to gastrointestinal AEs, patients may also gradually develop tolerance to GLP-1RA-related neuropsychiatric AEs with the prolonged application of GLP-1RAs. Clinicians need to dynamically assess patients’ neuropsychiatric side effects after clinical application of GLP-1RAs.

In the disproportionate analysis, statistically significant and potentially unexpected signal RORs were identified in olfactory nerve anomaly and sensory nerve disorder. Our research indicated that in the diabetic population, positive signals for olfactory nerve-related AEs were found in dulaglutide, liraglutide, and semaglutide, with semaglutide having the strongest signal; among antidiabetic medications, GLP-1RAs were also the only drug associated with olfactory AEs. In the obese population, only semaglutide had relevant signals, but the positive signals disappeared in the anti-obesity medicines. Of note, decreased olfactory acuity and olfactory dysfunction are found in the DM population. Some scholars believe that olfactory impairment can be an early risk marker for preclinical dementia in DM [41]. This may explain the positive signals of GLP-1RAs with olfactory abnormalities being stronger in DM than weight-loss populations. However, current research reckoned GLP-1RAs as improving olfactory impairment [41–44]. They suggest that the olfactory bulb is a brain region responsible for the first processing of olfaction, and it produces GLP-1 and has the distribution of GLP-1 receptors, indicating the potential mechanism of GLP-1RAs for olfactory improvement [43, 44]. Our findings were contrary to the previous mainstream views. However, given that there was still a strong signaling association between GLP-1RAs and olfactory abnormalities among antidiabetic medications, it was reasonable to be wary of GLP-1RA-induced olfactory abnormalities. Considered differently, could what we believed to be the mechanism by which GLP-1RAs ameliorate olfactory dysfunction also exacerbate olfactory dysfunction in some cases, serving as a double-edged sword?

In the diabetic population, dulaglutide, liraglutide, semaglutide, and tirzepatide were all found to be associated with sensory abnormalities, of which semaglutide having the strongest signal; among antidiabetic medications, GLP-1RAs were the only ones with a moderately positive signal. However, current studies generally agree that GLP-1RAs can alleviate pain hypersensitivity and restore peripheral neuropathy [45, 46]. Besides, DM often causes sensory abnormalities due to combinations of neuropathy or vasculopathy. For these reasons, when a diabetic patient complains of a new onset sensory abnormality, we tend to attribute this to complications of DM. But in the group of antidiabetic medications, GLP-1RAs were significantly correlated with sensory abnormality compared to other drugs, which may suggest that we should be vigilant for the involvement of GLP-1RAs in the sensory abnormality of a patient with emerging sensory abnormality in whom GLP-1RAs were being applied, especially in patients who achieved adequate blood glucose control. In the weight-loss population, semaglutide was the only GLP-1RA drug with weak signals; among anti-obesity medications, GLP-1RAs still had signals but weaker than naltrexone-bupropion, phentermine, and phentermine-topiramate. Given that obesity itself caused sensory abnormalities [47], we believed that the association between GLP-1RAs and sensory abnormalities in obese populations needed to be evaluated more cautiously, and subsequent, larger-samples and well-designed studies are needed for further exploration.

Headache and migraine are common side effects of drugs, which can reduce drug adherence and lower patients’ quality of life [48]. In the DM cohort, our study observed that all five GLP-1RAs were associated with headaches, dulaglutide, semaglutide, and liraglutide were linked to migraine, while this link was not seen in patients using these drugs for weight control. Also, among antidiabetic medications, only GLP-1RAs showed a positive signal for migraine and headaches, with liraglutide and semaglutide showing the strongest signals. Current research suggests a strong association between obesity and migraine [49, 50], while the relationship between DM and migraine remains controversial [51, 52]. Interestingly, despite higher doses of GLP-1RAs being used in weight loss populations and the apparent stronger connection between obesity and migraine, our findings indicate that headaches and migraines were more prominent in DM patients. DM patients are more likely to suffer from headaches due to hypoglycemia, diabetic ketoacidosis, cerebrovascular accidents, or other circumstances compared to the non-diabetic population. However, we only observed positive signals related to headaches in GLP-1RAs, rather than in other antidiabetic medications. Therefore, our finding suggested that headache may be associated explicitly with GLP-1RA treatment among the DM population. An article applied a network meta-analysis and found that GLP-1RA drugs significantly increased the risk of headache compared to insulin, thiazolidinediones, or placebo, with odds ratios of 1.34, 1.41, and 1.18, respectively [53]. According to the literature [53], GLP-1RAs cause headaches by increasing regional cerebral blood flow and decreasing blood pressure, resulting in the expansion of cerebral blood vessels and the stretch of peripheral nerves. In addition, GLP-1 receptors were widely distributed in the brain, suggesting that central action may also be one of the causes of headaches. It was worth noting that a case report suggested that GLP-1RA-induced nausea and vomiting might cause dehydration, leading to cerebral venous thrombosis and headache [54].

Depression, anxiety, suicide, or any other mental disorders are regarded as essential ADRs because they seriously affect the life quality of patients and even endanger life safety, and we observed weak signals among some GLP-1RAs. Regarding anxiety, we found that in patients with DM, semaglutide and exenatide were related to weak signals, and no similar signals were seen in weight-loss population. As for depression, only semaglutide had a weak positive signal in the DM population. Among other common antidiabetic medications, DPP-4 inhibitors were found with weak signals of anxiety and depression. Both DPP-4 inhibitors and GLP-1RAs belong to the category of incretin-based therapies, and although the action mechanisms of GLP-1RAs and DPP-4 inhibitors are different, their ultimate goal is the similar: to prolong the half-life of GLP-1 and increase its activity. This suggests that DPP-4 inhibitors may also influence psychoemotional responses through GLP-1RA-like effects. In terms of suicide, there was still a positive signal for semaglutide, and the signal seemed stronger for weight-loss than DM population. Potential associations between semaglutide and suicidal ideation have also been found in previous pharmacovigilance studies, and such associations were not observed in dulaglutide and exenatide [55, 56]. Among antidiabetic drugs, metformin showed a stronger suicide signal, which was in line with previous studies [55]. This indicated that more attention was paid to whether metformin increased the risk of suicide and that further studies were needed to investigate the relationships between metformin and suicide.

Semaglutide was associated with suicide more strongly in weight control than DM groups, which may be because obese people with larger doses of GLP-1RAs may lose more weight. Rapid weight loss can elicit significant emotional, biological, and psychological responses [32]. According to literature, GLP-1 can regulate neurotransmitter release (serotonin, dopamine, gamma-aminobutyric acid, and glutamate) to impact mood and psychology. GLP-1 receptors are found in emotion regulation areas such as the amygdala, dorsal raphe, and hippocampus. Additionally, GLP-1RAs can modulate CNS emotional responses by regulating taste and food-related reward [57–59]. Some studies suggested that frequent gastrointestinal reactions related to GLP-1RAs may lead to mental and psychological problems in patients [34], and the most significant gastrointestinal risks were observed in patients receiving semaglutide treatment [60]. This may explain why semaglutide had positive signals in almost all these neuropsychiatric abnormalities.

While we have seen positive suicide signals for semaglutide, particularly in the weight loss population, some real-world studies found that there was no relationship between GLP-1RAs and mental disorders [26, 37, 38, 61–64]; they even further pointed out that GLP-1RAs had a protective effect on depression and suicidal behaviors [61, 64, 65]. A pharmacovigilance study concluded that the combination of GLP-1RAs with other neuropsychiatric medications did not increase suicide risks [38]. On January 11, 2024, the FDA announced that no link was found between GLP-1RAs and the occurrence of suicidal thoughts or behaviors [66].

There are some limitations to this study. First, FAERS-based disproportionality analyses neither show causality nor quantify risks but only count the strength of the association. Then, in the FAERS database, it is not possible to know if patients had already comorbid psychiatric disorders and to assess all the co-prescriptions (that can also increase the risk of neuropsychiatric disorders). Finally, reporting bias, such as underreporting or overreporting due to varying levels of public awareness of medication side effects, especially in the context of the 78.6% of cases reported by non-healthcare professionals and our inability to differentiate between whether or not these reports were relevant to the litigation, could have introduced a potential for bias and affected our interpretation of the results. There are some strengths of this study. First, this study included a large number of cases of neuropsychiatric AEs related to GLP-1RAs, and second, the real data are a weakness in terms of data quality but also a strength as there are no “restrictions” on who receives the treatment, whereas randomized controlled trials have numerous exclusion criteria. Therefore, any conclusions drawn from pharmacovigilance analyses should be interpreted within the context of these constraints, and extensive prospective studies will need to be confirmed in the future.

## Conclusions

In this study, we found, for the first time, positive signals for GLP-1RAs with migraine, olfactory abnormalities, and sensory abnormalities; we also observed positive suicide signals for semaglutide, especially in weight loss population. Among the common GLP-1RAs, semaglutide had a relatively higher proportion of neuropsychiatric AEs, and essentially had positive and the strongest signals across these wide range of neuropsychiatric abnormalities. GLP-1RA-related neuropsychiatric AEs mostly reported within 30 days. Clinicians should continue to balance the benefits and risks associated with GLP-1RAs with a patient-centered approach, which can be followed up by improving baseline screening for diabetes- and obesity-related neuropsychiatric abnormalities, enhancing regular follow-up and increasing clinical vigilance for unusual neuropsychiatric manifestations.

## Supporting information

Lu et al. supplementary material Lu et al. supplementary material

## Data Availability

The datasets generated and/or analyzed during the current study are available in the U.S. FAERS database (https://fis.fda.gov/extensions/FPD-QDE-FAERS/FPD-QDE-FAERS.html). The code generated and/or analyzed in the current study is available from the corresponding author on reasonable request.

## Supplementary material

The supplementary material for this article can be found at http://doi.org/10.1192/j.eurpsy.2024.1803.
